# Individual and household-level predictors of health related quality of life among middle-aged people in rural Mid-east China: a cross-sectional study

**DOI:** 10.1186/1471-2458-14-660

**Published:** 2014-06-28

**Authors:** Jianfang Zhou, Xiaomei Ru, Norman Hearst

**Affiliations:** 1 Institute of Humanities and Social Sciences, Nanjing University of Posts and Telecommunications, Wenyuan Road 9, New Yadong District, 210023 Nanjing, China; 2 Chinese Family Planning Science and Technology Research Institute, Dahuisi 12, Haidian District, 10081 Beijing, China; 3 Departments of Family and Community Medicine and of Epidemiology and Biostatistics, University of California, 94143 San Francisco, CA, USA

**Keywords:** China, Household, HRQOL, Middle age, Rural

## Abstract

**Background:**

China has an enormous and growing middle-aged population. Little is known about health-related quality of life (HRQOL) for this group, especially in rural areas. We examined HRQOL and its individual and household predictors among middle-aged people in rural Mid-east China.

**Methods:**

HRQOL questionnaires and information about individual and household characteristics were collected from 428 subjects aged 45 to 65 in 12 villages in Mid-east China. We examined the eight dimensions of the SF-36 instrument, along with the Physical Component Summary (PCS) and Mental Component Summary (MCS) using a reference sample in Hong Kong for standardization. Individual and household predictors of PCS and MCS were examined by one-way ANOVA and binary logistic regression analysis.

**Results:**

Self-reported HRQOL was similar to that seen in middle-aged populations elsewhere. Based on univariate analyses, PCS differed by age, education, occupation, household per capita income, drinking water supply, and frequency of household members caring about each other; MCS differed by education, household per capita income, drinking water supply, and frequency of caring about each other. Individual and household-level factors accounted for 12.5% and 8.2% of the variance in PCS, respectively, and for 3.1% and 10.7% of the variance in MCS.

**Conclusions:**

HRQOL among middle-aged people in rural China appears similar to that observed elsewhere, and varies by income, education, and other factors. Household factors, particularly the extent to which household members care about each other, are significant predictors of physical and mental health. In addition to improving general socioeconomic conditions, efforts to improve HRQOL for middle-aged people in rural China need to focus on the family environment.

## Background

In modern society, rising standards of living and advances in public health and medical care have prolonged the average lifespan, while lower fertility rates have decreased the proportion of young people in the population [1]. Both of these trends have increased the proportion of middle-aged and older people in the population [2]. This has been particularly true in China. In 2010, 24.3% of China’s total population, or 324,327,480 people, were between 45 and 65 years old [3].

Health-related quality of life (HRQOL) is an individual's satisfaction or happiness with the dimensions of life insofar as they affect or are affected by "health" [4,5]. To date, a number of questionnaires have been developed to measure HRQOL and the 36-item Short Form Health Survey (SF-36) is the most commonly used [6,7]. The SF-36 has been adapted and applied in more than 40 countries as part of the International Quality of Life Assessment (IQOLA) Project [8]. Since Li first introduced and tested the version of the SF-36(v2) for use in China in 2002 [9] several subsequent studies have confirmed its high reliability and validity for use in the Chinese general population and among patients with chronic diseases [10-13].

Because health during middle age is important both in itself and as a predictor of health at older ages, knowing more about HRQOL among the middle-aged population is especially important in an aging society like China [14]. But very little is known about HRQOL among the middle-aged population of China. The limited available research indicates that: (1) HRQOL for this group is not very good [15-18]; (2) physical scores are higher than mental health scores in most studies [12,16,17], with the exception of one study in Shanghai [6]; and (3) HRQOL scores differed by sex, age, occupation and education [15-20]. However, none of these earlier studies was conducted in rural areas, where hundreds of millions of middle-aged Chinese people live and where socioeconomic conditions influencing HRQOL may differ from those in urban areas.

Prior studies in China have emphasized the individual factors influencing HRQOL, while the international literature on HRQOL demonstrates the importance of household factors. A large, longitudinal, nationally representative survey of British adults conducted in the 1990’s emphasized the importance of household membership and characteristics among the social factors affecting HRQOL [21]. One U.S. study found that household-level variables accounted for 4.5% and 26.1% of the total variance in self-reported individual physical and mental health status, respectively [22]. Another study of older adults in rural Vietnam found that people who were currently in married partnerships and from wealthier households reported better health [23]. This article analyzes new data collected in 2013 to describe HRQOL among people between 45 and 65 years old in an area of rural Mid-east China, examining both individual and household-level predictors.

## Methods

### Study sample

**Figure 1:**
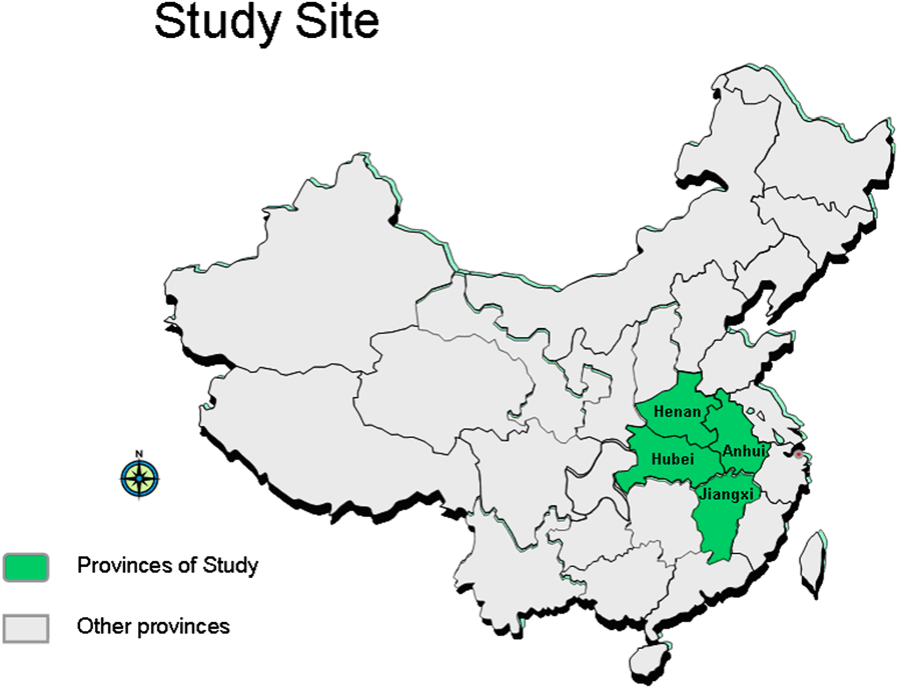
Study location.

In March 2013, we conducted a cross-sectional survey of eligible adults in 600 rural households in four counties in China: Zengdu in Hubei province, Nanle in Henan province, Huoshan in Anhui province, and Ji’an in Jiangxi province (see Figure 1). The sample sites were drawn based on convenience sampling from 12 participating counties in the Sino-Japanese technical cooperation project, “Infectious Diseases Prevention and Household Health Care (2011-2015) in Mid-east China.” Selection criteria for project sites included distance from the local airport (within 3 hours by bus) and relative per capita income level compared with the provincial average. As such, each site was typical for Mid-east China, which is less developed than China’s eastern seaboard.

We used a two-stage sampling method to select the surveyed families in the four sample counties. In the first stage, we selected three villages in each county by judgment sampling. Our goal was to select a representative sample of village environments, especially related to economic level and health service availability, based on the investigators’ previous knowledge of the study area. Chinese counties usually have a central city where the county government is located that has the biggest and best hospital in the county and that also serves as a commercial and cultural center. Typically, villages closer to the county center are richer and those farther away are poorer. To obtain a sample including a broad spectrum of village environments, we selected study villages based on their distance from the county center and per capita income in 2011. In each sample county, one village was selected that was near the county center and had a relatively high per capita income, another that was at an intermediate distance from the county center and had a per capita income similar to the county average, and a third that was farther away and relatively poor. In the second stage, we randomly selected 50 households in each of the twelve sample villages based on household lists from the local public security bureau.

We collected data from eligible adults in the 600 sampled households using two questionnaires. The first was a household questionnaire completed by an adult who was randomly selected among those at home at the time, and the second was an individual questionnaire administered to each adult household member. Household and individual questionnaires were linked by codes. Trained interviewers visited each household to distribute the questionnaires. If the participating adults were illiterate, they were interviewed verbally; if they could read and understand the written questionnaires, they completed the questionnaires by themselves. Due to no adult being at home during any of the four days of data collection, we missed 23 sample households.

This study was approved by the Nanjing College for Population Program Management Ethics Committee. All participants gave informed consent prior to inclusion in the study. No one refused to participate. While this may seem unusual by international standards, it is not surprising for research in rural China. Factors contributing to participation in this study included: (1) The interviewers were all local people. Villagers would not usually refuse an “insider” according to Chinese culture; (2) If the participants were busy at that time, the interviewer would make an appointment to return at another time; (3) Participants were given a small gift (about $1) for their time.

This article presents findings for 428 surveyed adults who were between 45 and 65 years old from 320 households; 87.2% of these study subjects completed the written questionnaires by themselves. An additional 31 subjects in households that we contacted in the age group that would have been eligible for the study were excluded because they were not in their village during the data collection period; 28 of these were working outside of their counties.

### Variables

#### ***Questionnaire development and testing***

This study included two questionnaires. One was a questionnaire of household factors designed by the investigators. The other was an individual questionnaire including personal factors and health status. We used SF-36(v2) to measure health status. Other questions covered factors potentially related to HRQOL based on published reports and the investigators’ knowledge of the life of middle-aged people in rural China. Both questionnaires were pretested among 30 households in rural Nanle County in Henan Province of China.

#### ***Health***

This study used the SF-36(v2) to measure participants’ HRQOL. The SF-36 is a 36-item questionnaire measuring HRQOL and can be administered in 5 to 10 minutes. The response format of the items varies from two to six response categories. One item measures current health status compared with one year earlier, while the remaining 35 items yield an eight-dimensional profile of physical and mental health during the past four weeks. The eight SF-36 dimensions—physical functioning (PF), role limitations due to physical health problems (RP), bodily pain (BP), general health (GH), vitality (VT), social functioning (SF), role limitations due to emotional health problems (RE), and mental health (MH)—were individually scored and also combined into two categories representing physical functioning and wellbeing and emotional wellbeing: the Physical Component Summary (PCS), including physical functioning, role limitations due to physical health problems, bodily pain, and general health; and the Mental Component Summary (MCS), including vitality, social functioning, role limitations due to emotional health problems, and mental health. Scores of the 8 individual health dimensions and of the PCS and MCS were calculated according to the SF36 (v2) manual. Because a full Chinese SF36 (v2) normalized model is not yet available, we used the results of an earlier Chinese study as the normal model to calculate PCS and MCS [24].

#### ***Individual characteristics***

Based on previous studies [15-20], this study collected data on respondents’ date of birth (this was converted into age in years and analyzed as a continuous variable); sex; highest level of education (illiterate, elementary school, junior middle school, senior middle school, college); current marital status (married vs. not married); and current occupation (farmer, worker, unemployed/retired).

#### ***Household factors***

We constructed questions to assess household factors based on variables that seemed most relevant to the local context based on the investigators’ knowledge of these areas. Participants were asked about the number of people in their household, whether they lived with people other than their spouse; their town or village of residence; the size of their home in square meters; the kind of water their household members drink daily (safe water, such as tap water/pure water, or unsafe water, such as river, well or spring water); earned household income during the prior year; and if their household members always, often, seldom, or never cared about each other. We included the question regarding household members caring about each other because this has been a popular topic in Chinese mass media recently.

### Analysis

**Figure 2:**
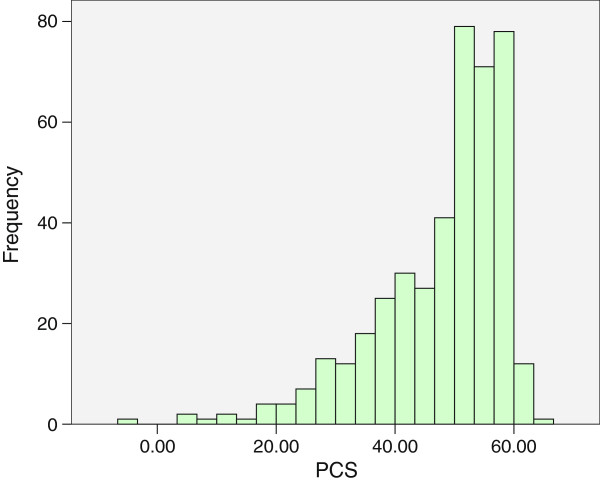
Distribution of PCS scores among 428 middle-aged residents of 12 rural villages in Mid-east China in 2013.

**Figure 3:**
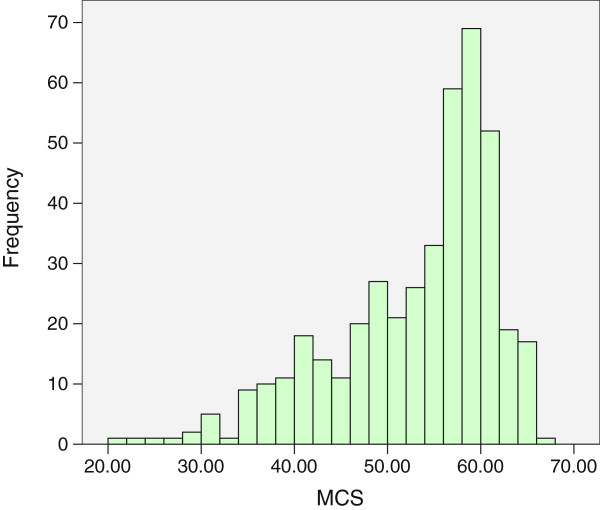
Distribution of MCS scores among 428 middle-aged residents of 12 rural villages in Mid-east China in 2013.

We used Epidata 3.2a to record responses and analyzed them using SPSS 17.0. Interviewers and investigators double-checked all questionnaires in the field, so no questionnaire items were missing for more than 1% of respondents. In the few cases where this did occur, we substituted the mode value (for qualitative variables) or mean value (for quantitative variables).After describing the distributions of the variables of interest, we estimated the bivariate associations of the PCS and MCS scores with each individual and household-level characteristic using one-way ANOVA. Due to the relatively small samples and the left-skewed distribution of PCS and MCS scores (shown in Figures 2 and 3 respectively), binary logistic regression was then applied to analyze the associations of PCS and MCS scores with their potential individual and household-level predictors in a multivariate model. For this analysis, we dichotomized PCS and MCS as lower than 50 (valued as “0”) versus greater than or equal to 50 (valued as “1”). For the binary logistic regression analysis, the potential related factors as independent variables were entered in 2 blocks: block 1 included only individual-level factors, while block 2 included household-level factors. The results of effects (measures of association) are shown as β, OR (95% CI) and significance (P value). P < 0.05 was considered significant.

Many of the households sampled had no members in the age group of this study. Others had more than one eligible member included in the study. Insofar as people in the same household are more likely to have similar HRQOL than people in different households, this may violate the assumption of independence in our multivariate model. On the other hand, excluding multiple eligible subjects in the same household from the study would have resulted in underrepresentation of married persons in our sample. To examine this, we conducted a separate multivariate analysis excluding more than one participant from the same household (remaining N = 320). The results of this analysis (not presented) were almost identical to the analysis of all 428 subjects, except for wider confidence intervals due to the smaller sample size. For simplicity, we present only our analysis of all 428 subjects.

## Results

### Individual and household characteristics

As seen in Table 1, the mean age of respondents was 54 years, and nearly half were female. Most were literate and 43% had middle school or higher diplomas. More than half were farmers. All but 6% of respondents were currently married, and half lived with people other than their spouses. Their mean household per capita living space was 46.06 m^2^, and 49% used tap water/pure water as daily drinking water. Average household per capita income was 8098 ¥ (about 1200 US$). Seventy percent of respondents reported that their household members always cared about each other.

**Table 1:** Individual and household characteristics of 428 middle-aged residents of 12 rural villages in Mid-east China in 2013

	**Characteristic**	**Percent of sample**
Individual characteristics	Sex	
	Male	51.7
	Female	48.3
	Age (years)	
	45-50	31.2
	50-55	24.0
	55-60	25.9
	60-65	18.9
	(Mean = 54.1)	
	Educational attainment	
	Illiterate	15.2
	Elementary school	42.2
	Junior middle school	32.9
	Senior middle school or higher	9.8
	Occupation	
	Farmer	56.4
	Workers	18.4
	Unemployed/retired/house worker	25.2
Household characteristics	Marital status	
	With spouse	94.2
	Without spouse	5.8
	Lives with others besides spouse	
	Yes	50.0
	No	50.0
	Household per capita living space (m^2^)	
	<20	11.8
	20-40	44.5
	40-60	24.7
	≥ 60	19.1
	(Mean = 46.1)	
	Household per capita income (￥)	
	<2000	6.3
	2000-4999	21.9
	5000-9999	35.4
	10000-14999	25.9
	≥15000	10.5
	(Mean = 8496)	
	Daily drinking water quality	
	Safe	49.2
	Unsafe	50.8
	Frequency of household members caring about each other	
	Always	70.4
	Often	24.7
	Seldom	4.9
	Never	0.0

### Health status

Scores for the 8 individual dimensions of health ranged from 63.46 for general health to 89.18 for physical functioning. Average PCS and MCS scores of participants were 46.97 and 53.11 respectively, with scores concentrated at the higher side (more than 50) of the distributions. In Table 2, we compare our results to those of two other Chinese studies. When calculating PCS and MCS scores, the Hong Kong study was used as the normal model for all three [6,24].

**Table 2:** Comparison of SF-36 dimension scores among studies in Hong Kong, Shanghai and rural Mid-east China

	**Rural Mid-east China**		**Shanghai **[6]		**Hong Kong **[24]	
**Sample**	**428 subjects aged from 45 to 65 years**		**919 subjects aged from 18 to 77 years**		**2410 subjects aged more than 18 years**	
**Survey year**	**2013**		**2005-2006**		**1998**	
	**Mean**	**SD**	**Mean**	**SD**	**Mean**	**SD**
PF	89.2	15.2	89.7	14.8	91.8	12.9
RP	74.5	37.8	93.8	22.6	82.4	31.0
BP	73.2	19.1	94.6	13.8	84.0	21.9
GH	63.5	22.0	68.8	19.4	56.0	20.2
VT	73.9	15.3	71.8	18.3	60.3	18.7
SF	88.8	17.4	94.3	12.1	91.2	16.5
RE	77.5	34.5	95.1	20.6	71.7	38.4
MH	74.2	15.8	81.8	14.7	72.8	16.6
PCS	47.8	11.0	Female 52.5	Female 5.9	50.0	10.0
			Male 53.4	Male 5.6		
MCS	53.1	8.6	Female 54.4	Female 6.3	50.0	10.0
			Male 54. 5	Male 6.1		

Physical Component Summary (PCS) and Mental Component Summary (MCS) represent physical functioning and wellbeing and emotional wellbeing, respectively. PCS and MCS were calculated using the Hong Kong study as the normal model. For the Shanghai study, MCS and PCS were only available separately by sex.

Table 3 presents PCS and MCS scores by respondents’ individual and household characteristics. One-way ANOVA analysis showed that: (1) PCS differed by age, education, occupation, household per capita income, drinking water supply, and frequency of household members caring about each other; and (2) MCS scores differed by education, household per capita income, daily drinking water supply, and frequency of caring about each other.

**Table 3:** Physical and mental health related quality of life by individual and household characteristics among 428 middle-aged residents of 12 rural villages in Mid-east China in 2013

**Variables**	**Values**	**PCS**			**MCS**		
		**Mean**	**SD**	**P Value**	**Mean**	**SD**	**P Value**
Sex	Male	48.68	10.93	0.093	53.20	8.82	0.828
	Female	46.89	11.09		53.02	8.53	
Age	45-50 years	50.79	8.21	< .001	53.91	8.60	0.277
	50-55 years	49.12	9.44		53.46	8.10	
	55-60 Years	46.78	10.92		52.94	8.49	
	60-65 years	42.34	14.61		51.59	9.58	
Education	Illiterate	44.64	13.57	< .001	51.56	9.25	0.002
	Elementary school	46.15	11.35		51.82	9.38	
	Junior middle school	50.72	8.87		54.89	7.48	
	Senior middle school/higher	49.61	9.49		55.11	6.75	
Occupation	Farmer	48.31	10.84	0.005	52.96	8.68	0.337
	Workers	49.91	8.90		54.35	7.86	
	Unemployed/retired/house worker	44.94	12.37		52.54	9.13	
Marital status	With spouse	47.97	10.82	0.101	53.22	10.33	0.312
	Without spouse	44.24	13.89		51.41	8.55	
Stay with others except spouse	Yes	48.13	11.48	0.461	53.14	8.32	0.916
	No	47.34	10.60		53.06	9.00	
Household per capita living space (m^2^)	less than 20	48.86	9.67	0.652	54.64	7.76	0.449
	20-40	47.03	11.94		53.00	8.93	
	40-60	48.04	10.66		52.33	7.91	
	More than 60	48.37	10.19		53.52	9.29	
Household per capita income (¥)	Less than 2000	45.53	12.93	0.008	51.81	9.39	< .001
	2000-4999	44.70	13.38		50.63	9.26	
	5000-9999	48.03	10.86		52.13	8.98	
	10000-14999	50.02	8.31		55.83	7.02	
	More than 15000	48.98	9.70		55.82	7.18	
Daily drinking water	Safe water	48.97	8.80	0.024	54.68	7.38	< .001
	Others	46.58	12.75		51.60	9.50	
Frequency of caring about each other	Always	49.00	9.98	< .001	53.93	8.11	< .001
	Often	46.53	10.75		52.01	9.32	
	Seldom	36.14	18.12		46.95	10.03	

The data in this table are from one-way ANOVA analysis.

### Binary logistic regression analysis of health-related household factors

Tables 4 and 5 show the results of binary logistic regression analysis with PCS and MCS as dependent variables. For PCS, in the block 1 model (with only individual characteristics as potential predictors), R squared equaled 0.125 and the model was significant (p < 0.001). After adding household factors, the R squared of the block 1 and 2 model increased to 0.207. The household factors thus explained 8.2% of PCS variance. Household per capita income, drinking water and frequency of household members caring about each other were significantly associated with PCS. Those who were from families with higher income, drinking safe water, and always caring about each other had better PCS scores.

**Table 4:** Binary logistic regression analysis of predictors of PCS among 428 middle-aged residents of 12 rural villages in Mid-east China in 2013

**Variable (reference value)**	**Variable’s value**	**Block 1 model**			**Block 1 and 2 model**		
		**β**	**OR(95% CI)**	**Sig.**	**β**	**OR(95% CI)**	**Sig.**
Sex(male)	Female	-0.301	0.461-1.189	0.214	-0.332	0.442-1.164	0.178
Age (per year)	---	-0.066	0.899-0.975	0.002	-0.059	0.904-0.963	0.006
Occupation (unemployed)	Agriculture	0.373	0.863-2.446	0.180	0.412	0.879-2.596	0.135
	Workers	0.414	0.742-3.088	0.255	0.310	0.656-2.831	0.407
Education (illiterate)	Elementary school	-0.242	0.429-1.436	0.432	-0.284	0.402-1.410	0.376
	Junior middle school	0.494	0.832-3.233	0.153	0.510	0.811-3.417	0.165
	Senior middle school	0.387	0.598-3.628	0.400	0.382	0.584-3.676	0.416
Marital status (with spouse)	Without spouse	-0.182	0.348-1.998	0.683	-0.163	0.336-2.150	0.731
Lives with others except spouse (yes)	No				-0.211	0.521-1.260	0.350
Household per capita income (per 1000 ¥)	---				0.061	1.007-1.122	0.026
Household per capita living space (per m^2^)	---				-0.001	0.994-1.004	0.616
Drinking water supply (unsafe water)	Safe water				0.472	1.239-3.316	0.041
Frequency of household members caring about each other(seldom)	Always				1.184	1.138-9.384	0.028
	Often				0.817	0.754-6.792	0.145

Block 1 includes only individual-level factors; block 2 also includes household-level factors. For the block 1 model: R squared = 0.125, Model Sig. <0.001; for the block 1 and 2 model: R squared = 0.207, Model Sig. <0.001.

**Table 5:** Binary logistic regression analysis of predictors of MCS among 428 middle-aged residents of 12 rural villages in Mid-east China in 2013

**Variable (reference value)**	**Variable’s value**	**Block 1 model**			**Block 1 and 2 model**		
		**β**	**OR(95% CI)**	**Sig.**	**β**	**OR(95% CI)**	**Sig.**
Sex(male)	Female	-0.132	0.536-1.432	0.598	-0.252	0.464-1.302	0.339
Age (per year)	---	-0.020	0.940-1.023	0.363	-0.010	0.947-1.036	0.671
Occupation (unemployed)	Agriculture	0.108	0.652-1.903	0.694	0.308	0.768-2.413	0.292
	Workers	0.026	0.489-2.155	0.944	-0.242	0.359-1.716	0.544
Education (illiterate)	Elementary school	0.008	0.547-1.856	0.980	-0.226	0.416-1.532	0.498
	Junior middle school	0.440	0.767-3.144	0.222	0.146	0.540-2.480	0.707
	Senior middle school	0.602	0.693-4.808	0.223	0.399	0.544-4.085	0.438
Marital status (with spouse)	Without spouse	-0.193	0.352-1.935	0.658	-0.211	0.320-2.050	0.656
Lives with others except spouse (yes)	No				-0.485	0.384-0.988	0.044
Household per capita income (per 1000 ￥)	---				0.156	1.098-1.245	<0.001
Household per capita living space (per m^2^)	---				-0.003	0.992-1.002	0.285
Drinking water supply (unsafe water)	Safe water				0.581	1.111-2.879	0.017
Frequency of household members caring about each other(seldom)	Always				0.963	1.087-6.952	0.043
	Often				0.357	0.516-3.959	0.493

Block 1 includes only individual-level factors; block 2 also includes household-level factors. For the block 1 model: R squared = 0.031, Model Sig. = 0.320; for the block 1 and 2 model: R squared = 0.138, Model Sig. <0.001.

For MCS, R squared was 0.031 in the block 1 model and increased to 0.138 in the block 1 and 2 model. Household factors thus explained 10.7% of MCS variance. MCS scores were higher among respondents with higher household per capita incomes, safe drinking water, and who reported a higher level of family members caring about each other.

## Discussion and conclusion

Compared to the Shanghai study, the average RP, BP, GH, SF, RE, and MH scores of this sample were lower by more than 5 points. Compared to the Hong Kong sample, the average RP, BP, RE, and MH scores of this sample were lower by more than 5 points but their VT and RE scores were higher by more than 5 points. The findings for our sample of middle-aged people in rural Mid-east China indicate that they had better mental health (53.1) but worse physical health (47.8) than the Hong Kong sample, for which PCS and MCS were both 50 because this was the reference standard. Both mental health and physical health were worse in our sample than in the Shanghai sample and HK sample [6,25]. Their scores in 8 dimensions were similar to scores of a Swedish middle-aged population [26] but different from scores of Australian middle-aged women whose PF, VT and SF scores (PF = 85.08, VT = 58.08, SF = 81.38) were lower and GH score (71.90) was higher [27].

This study also found that the respondents’ MCS scores were higher than their PCS scores, in contrast to findings from most previous studies in China [12,16,17], which focused on urban areas, but similar to those from several earlier studies in Shanghai [6], Japan [28], India [29] and Australia [30] that included some participants from rural areas. The Shanghai study also showed that whether participants were from rural areas was significantly related to RP, BP and MH [6] with people in urban areas reporting higher stress levels than those in rural areas. Three comparison studies in China showed that urban people had lower subjective well-being and higher stress than rural people [31-33]. A study among Chinese middle-aged intellectuals showed that stress was one of most important HRQOL-related factors [18].

Our results indicate various factors associated with HRQOL among the rural middle-aged population in Mid-east China. Age, educational level and economic status were strongly related to HRQOL. Increasing age was associated with deteriorating physical and mental health; higher education was associated with better physical and mental health, perhaps reflecting increased knowledge about health matters; and lacking a paid job and low household per capita income were associated with poorer mental health. These findings are consistent with previous studies conducted in Singapore, Pakistan, India, Mexico and elsewhere [6,29,34-38]. This research did not find significant sex differences in HRQOL. This was different from studies of Chinese middle-aged intellectuals [15-18] but similar to other studies in Shanghai and Hong Kong [6,25]. In the studies of intellectuals, women had lower PCS and MCS scores than men. This may due to their higher level of stress in their work and daily lives. Under traditional Chinese culture, these women may need to work harder than men to obtain the same rewards at work while also being mainly responsible for housework. Rural middle-aged women, on the other hand, usually only do housework and a limited amount of farm work and may thus be under less stress.

Of particular interest in this study was how individual and household-level characteristics related to HRQOL. Binary logistic regression analysis showed similar results to earlier studies from Vietnam and the United States [22,23]. Household-level characteristics explained a substantial amount of the variance in HRQOL among middle-aged people in Mid-east China. Individual-level characteristics explained more PCS variance (12.5%) than MCS (only 3.1%). Household-level characteristics explained more MCS variance (10.7%) than PCS variance (8.2%). The percent of variance explained by household-level characteristics was different in these three studies, probably due to differing samples, methodology and cultural factors. But all found household income to be an important household-level predictor.

This study also found two other novel household-level predictors of HRQOL: drinking water supply and frequency of family members caring about each other. Middle-aged people whose household used safe water and cared about each other more frequently had higher PCS and MCS scores. Safe drinking water is currently a very popular topic in China. People are worried about water safety. Our research found that only half of rural families used tap water or pure water although the Chinese government had promised that rural tap water supply coverage would reach 75% by 2010 [39]. In our sample, lower household per capita income families had a higher proportion of not using safe water (P = 0.001 by Chi-square). Being poor and using unsafe water are thus double negative factors for some people. These results support the importance of promoting safe tap water. Pipes not only need to reach every household. The pipes must always contain safe water, and the service must be affordable.

Caring about each other more frequently was also a positive factor for health of this middle-aged population. The explanation for this result may indicate: (1) If household members care about each other more, they will be happier and adjust to problems more quickly; (2) Good mental health may be an important positive factor for physical health; (3) If they care about each other more, other household members will have more chance to know about each other’s health, to provide support, and to encourage appropriate medical treatment.

This study has limitations. The cross-sectional nature of data limited our ability to understand causal mechanisms. Participants with low educational level may not understand SF36 as well, leading to inaccurate health assessment. We do not know the accuracy of responses to items such as income. Also, we used both verbal and written responses in this survey; whether there is a difference between these two data collection methods is unknown. Although SF36 has been validated elsewhere, more work is needed to determine how well it correlates with objective health status in this population. Although we believe our study villages to be fairly typical of rural Mid-east China, they were a judgment sample selected to give a broad range of villages. It was not possible to weight our sample to give results that would necessarily generalize to the three provinces studied, let alone to the rest of rural China. This study also excluded 31 targeted subjects because they were not at home during the data collection. Most of these were working outside the province, suggesting that the HRQOL scores of these “healthy workers” may have been higher had they been included, thus producing a downward bias on average scores. Despite these limitations, these results are important because this is the first study of HRQOL in middle-aged rural Chinese, a group larger than the entire population of all but a few countries.

In conclusion, this study provides cross-sectional evidence of the pattern of self-reported health status among a middle-aged population in rural Mid-east China. The findings revealed problems in terms of equity in health with some groups reporting better physical and mental health than others. We also found that household factors, including household per capita income, drinking water supply and frequency of family members caring about each other, were strong predictors of HRQOL. According to these results, health inequality among middle-aged rural Chinese might be reduced by increasing education, income, and accessibility of safe water. The importance of a household environment in which people always care about each other appears to be fundamental.

## Competing interests

All authors declare that they have no competing interests.

## Authors’ contributions

JZ was principal investigator of this study and contributed to conception and design, acquisition of data, analysis and interpretation of data, and writing the manuscript. XR contributed to conception, design, and acquisition of data. NH contributed to analysis and interpretation of data and writing the manuscript. All authors read and approved the final manuscript.

## Authors’ information

JZ is an associate professor of Social Medicine and Population Management at the Humanities and Social Sciences Institute in Nanjing University of Posts and Telecommunications. XR is a health project management expert in the Chinese Family Planning Science and Technology Research Institute. NH is a physician/epidemiologist and a professor of Family and Community Medicine and of Epidemiology and Biostatistics at the School of Medicine of the University of California, San Francisco.

## Pre-publication history

The pre-publication history for this paper can be accessed here:

http://www.biomedcentral.com/1471-2458/14/660/prepub
